# Melatonin Promotes In Vitro Maturation of Vitrified-Warmed Mouse Germinal Vesicle Oocytes, Potentially by Reducing Oxidative Stress through the Nrf2 Pathway

**DOI:** 10.3390/ani11082324

**Published:** 2021-08-06

**Authors:** Shichao Guo, Jinyu Yang, Jianpeng Qin, Izhar Hyder Qazi, Bo Pan, Shengqin Zang, Tianyi Lv, Shoulong Deng, Yi Fang, Guangbin Zhou

**Affiliations:** 1 Farm Animal Genetic Resources Exploration and Innovation Key Laboratory of Sichuan Province, College of Animal Science and Technology, Sichuan Agricultural University, Chengdu 611130, China; daxiaopang@outlook.com (S.G.); yangjinyu19960710@163.com (J.Y.); qjp9890@163.com (J.Q.); bopan1992@163.com (B.P.); zsq820712921@163.com (S.Z.); lty_0211@163.com (T.L.); 2 Department of Veterinary Anatomy & Histology, Shaheed Benazir Bhutto University of Veterinary and Animal Sciences, Sindh, Sakrand 67210, Pakistan; vetdr_izhar@yahoo.com; 3 Institute of Laboratory Animal Sciences, Chinese Academy of Medical Sciences and Comparative Medicine Center, Peking Union Medical College, Beijing 100021, China; dengshoulong@cnilas.org; 4 Jilin Provincial Key Laboratory of Grassland Farming, Northeast Institute of Geography and Agroecology, Chinese Academy of Sciences, Changchun 130102, China

**Keywords:** vitrification, receptors, antioxidants, reactive oxygen species, Nrf2

## Abstract

**Simple Summary:**

Cryopreservation of oocytes can cause high oxidative stress, reduce the quality of vitrified-warmed oocytes, and seriously hinder the application of oocyte cryopreservation technology in production and medicine. In this work, we found for the first time that melatonin can exert antioxidant effects through receptors and regulate the Nrf2 antioxidant pathway to respond to oxidative stress of vitrified-warmed oocytes, thereby improving both oocyte quality and the potential for subsequent development. The results illustrated the molecular mechanism of melatonin’s antioxidant effect in vitrified-warmed oocytes and provided a theoretical basis for the application of melatonin in the cryopreservation of oocytes. These findings are of great significance for the further application of oocyte cryopreservation technology to production and assisted reproduction in the future.

**Abstract:**

Previously it was reported that melatonin could mitigate oxidative stress caused by oocyte cryopreservation; however, the underlying molecular mechanisms which cause this remain unclear. The objective was to explore whether melatonin could reduce oxidative stress during in vitro maturation of vitrified-warmed mouse germinal vesicle (GV) oocytes through the Nrf2 signaling pathway or its receptors. During in vitro maturation of vitrified-warmed mouse GV oocytes, there were decreases (*p* < 0.05) in the development rates of metaphase I (MI) oocytes and metaphase II (MII) and spindle morphology grades; increases (*p* < 0.05) in the reactive oxygen species (ROS) levels; and decreases (*p* < 0.05) in expressions of Nrf2 signaling pathway-related genes (*Nrf2*, *SOD1*) and proteins (Nrf2, HO-1). However, adding 10^−7^ mol/L melatonin to both the warming solution and maturation solutions improved (*p* < 0.05) these indicators. When the Nrf2 protein was specifically inhibited by Brusatol, melatonin did not increase development rates, spindle morphology grades, genes, or protein expressions, nor did it reduce vitrification-induced intracellular oxidative stress in GV oocytes during in vitro maturation. In addition, when melatonin receptors were inhibited by luzindole, the ability of melatonin to scavenge intracellular ROS was decreased, and the expressions of genes (*Nrf2*, *SOD1*) and proteins (Nrf2, HO-1) were not restored to control levels. Therefore, we concluded that 10^−7^ mol/L melatonin acted on the Nrf2 signaling pathway through its receptors to regulate the expression of genes (*Nrf2*, *SOD1*) and proteins (Nrf2, HO-1), and mitigate intracellular oxidative stress, thereby enhancing in vitro development of vitrified-warmed mouse GV oocytes.

## 1. Introduction

Oocyte cryopreservation has been used in genetic selection, the preservation of germplasm resources and scientific research [1,2]. More importantly, oocyte cryopreservation, combined with advanced biotechnologies, can avoid some of the ethical restrictions inherent in human-assisted reproduction techniques [3,4,5]. However, vitrification and warming procedures inflict cryodamage in oocytes and affect their cell membrane, organelles, DNA, and other structures [6,7,8]. Mitochondrial damage also causes the excessive accumulation of reactive oxygen species (ROS), resulting in high oxidative stress which will further damage mitochondria and other structures, affect cell signal transduction, and decrease developmental potential of vitrified-warmed oocytes [6,9]. Therefore, reducing the cellular oxidative stress can improve the quality of vitrified-warmed oocytes.

The NF-E2-related factor 2/Antioxidant response element (Nrf2/ARE) pathway, one of the important antioxidant response pathways in cells, is regulated by the active factor Nrf2 [10]. Normally, Nrf2 binds to Kelch-like ECH-associated protein-1 (Keap1) in the cytoplasm and is inactive. However, when intracellular oxidative stress increases, electrophiles and quinones bind to the cysteine residues of Keap1, resulting in conformational changes and the dissociation of Nrf2, enabling it to enter the nucleus, bind to the ARE sequence, and induce the expression of downstream antioxidant proteins [11], including Heme Oxygenase-1 (HO-1), Recombinant Glutamate Cysteine Ligase, and Modifier Subunit (GCLM) and Superoxide Dismutase (SOD) [12,13], with important roles in anti-oxidation and anti-apoptosis [14,15,16]. Cryopreservation can significantly decrease the expression of Nrf2 in porcine oocytes [17], contributing to increased oxidative stress in vitrified-warmed oocytes. However, it remains unclear whether cryopreservation can impact the Nrf2 signaling pathway and related proteins during in vitro maturation of mouse GV oocytes. Additionally, the possible relationship between changes in expression of these proteins and ROS-inflicted oxidative stress remains to be explored.

Melatonin (MT), an important antioxidant, can regulate the Nrf2 signaling pathway in cells [18]. Through the Nrf2 signaling pathway, melatonin, can antagonize oxidative injury induced by manganese (Mn) in the striatum of mice [19]; protect against early brain injury in a subarachnoid hemorrhage model in mice [20]; treat experimental allergic encephalomyelitis in mice [21]; attenuate acute kidney ischemia/reperfusion injury in diabetic rats [22]; prevent lipopolysaccharide- (LPS) induced epithelial–mesenchymal transition in human alveolar epithelial cells [23]; promote porcine embryo development [24]; and inhibit oxidative stress and apoptosis in cryopreserved ovarian tissues [25]. Although previously it has been shown that melatonin can reduce excessive ROS in oocytes induced by cryopreservation in mice [26,27,28,29], bovine animals [30], and humans [31], the underlying mechanism through which melatonin might regulate the Nrf2 signaling pathway remains to be elucidated.

Melatonin receptors, MT1 (MEL-1A) and MT2 (MEL-1B), are mainly distributed on the cell membrane [32,33,34,35]. Melatonin can have an antioxidant role in cells through receptors, such as by protecting against cisplatin-induced ovarian damage in mice [36] and delaying the fertility decline in female animals [37]. Melatonin can also regulate the intracellular Nrf2 signaling pathways through receptors, preventing the senescence of canine adipose-derived mesenchymal stem cells [38] and promoting the integrity of the blood–brain barrier in methamphetamine-induced inflammation in the primary microvascular endothelial cells of rat brains [39]. In preliminary studies, we determined that there were melatonin receptors on the membrane during in vivo maturation of mouse GV oocytes. Melatonin can reduce the oxidative stress level during the in vitro maturation of vitrified-warmed GV oocytes, but it remains to be studied whether this role is performed through its receptors.

It has been reported that Brusatol, a quassinoid from the seeds of *Brucea sumatrana*, can specifically inhibit the transcription and translation of the Nrf2 protein and can inhibit the function of Nrf2 pathway [40,41]. Luzindole (N-acetyl-2-benzyltryptamine) is considered an antagonist of melatonin receptors and is usually employed to study the signaling pathways involved in the action of melatonin in cells [42,43]. Therefore, our objective was to explore whether melatonin can reduce oxidative stress during in vitro maturation of vitrified-warmed mouse GV oocytes through the Nrf2 signaling pathway or its receptors.

## 2. Materials and Methods

Unless otherwise stated, all chemicals were commercially available from Sigma-Aldrich (St. Louis, MO, USA). The experimental mice were maintained and handled in agreement with the requirements of the Animal Ethical and Welfare Committee (AEWC) of Sichuan Agricultural University, as well as in accordance with the relevant laboratory animal management regulations (Ministry of Science and Technology of China, Beijing, June 2004) and with the approval of the Animal Health and Use Committee of Sichuan Agricultural University College of Animal Science and Technology (No. DKYB20081003).

### 2.1. Oocyte Collection

Female ICR mice (*n* = 298), 8–10 wks old, were purchased from Chengdu Dashuo Experimental Animal Co., Ltd. and maintained at 18–25 °C and 50–70% humidity, with 14 h of light and 10 h of darkness. After a 2-wk adaptation period, each mouse was intraperitoneally injected with 10 IU pregnant mare serum gonadotropin (PMSG, NingBo Second Hormone Factory, Ningbo, China). After 44–48 h, the mice were subjected to a sudden death by separating the neck from the base of the skull. Ovaries were taken out, placed in a 37 °C M2 solution, sliced under the stereomicroscope with syringe needles, and GV oocytes with obvious germinal vesicles were selected for experiments.

### 2.2. Oocyte Vitrification and Warming

Open-pulled straws (OPS) were prepared as described [26,44]. In brief, the straws (0.25 mL) were heat-softened and pulled manually, to obtain straws approximately 3 cm long, 0.10 mm inner diameter, and 0.15 mm outer diameter.

Vitrification solutions were (1) 10% dimethylsulfoxide (DMSO) and 10% ethylene glycol (EG) in phosphate-buffered saline (PBS) medium (referred as ED), and (2) 15% EG, 15% DMSO, 300 g/L Ficoll, 0.5 mol/L sucrose and 3 g/L bovine serum albumin (BSA) in PBS medium (referred as EDFS30).

Oocytes were vitrified by the OPS method. Firstly, the oocytes were equilibrated in ED for 30 s, and then transferred to EDFS30 for 25 s. Finally, the oocytes were precisely arranged at the end of the OPS (8–10) and quickly dropped into liquid nitrogen. For warming, the OPS was removed from liquid nitrogen and the end was quickly placed in warming solution (0.5 mol/L sucrose solution) equilibrium for 5 min, then transferred to M2 solution and rinsed three times. All manipulations were carried out at 37 °C on a temperature control stage attached to a stereomicroscope (SMZ1500, Nikon, Tokyo, Japan).

### 2.3. Oocyte Culture and In Vitro Maturation

**Figure 1:**
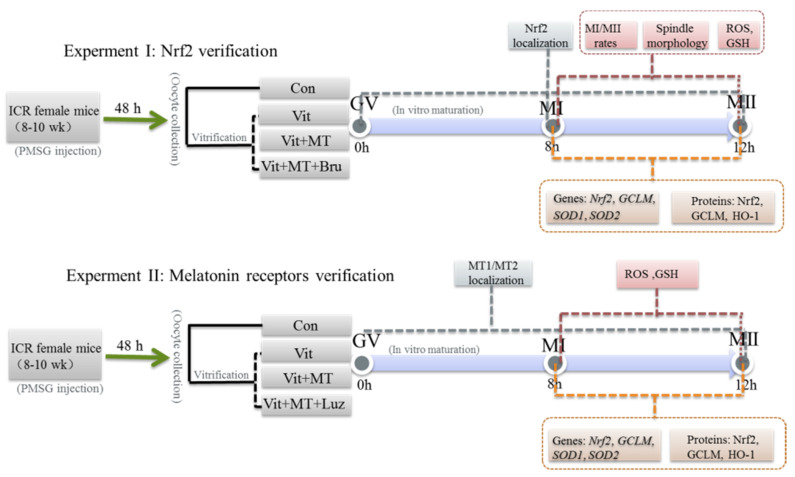
A flowchart of the experimental design. Notes: Brusatol, Nrf2 protein inhibitor; luzindole, Melatonin receptors inhibitor.

The GV oocytes were randomly assigned into following groups. Control group (Con): fresh oocytes matured directly in vitro; vitrification group (Vit): oocytes were vitrified and then matured in vitro; vitrification + melatonin group (Vit + MT): on the basis of vitrification group, 10^−7^ M melatonin [26] was added to the warming and maturation solutions (M16); vitrification + melatonin + brusatol group (Vit + MT + Bru): on the basis of vitrification group, 10^−7^ M melatonin was added to the warming and maturation solutions, and 50 nM Brusatol (Nrf2 inhibitor) [24,45] was added to the maturation solution; and vitrification + melatonin + luzindole group (Vit + MT + Luz): on the basis of vitrification group, 10^−7^ M melatonin was added to the warming and maturation solutions, and 10^−7^ M luzindole (Melatonin receptors inhibitor) [46] was added to the maturation solution. Then, fresh and vitrified oocytes were rinsed and placed in M16 medium. After 8 h, oocytes were stained with DAPI (Vector Laboratories Inc., Burlingame, CA, USA), and the chromosomes that were arranged in an orderly manner on the equatorial plate under a fluorescence microscope (BX53F, OLYMPUS, Tokyo, Japan) were in the MI stage. Similarly, 14 h later, the oocytes of each group were observed under a stereomicroscope, and the first polar body (PB-I) was extruded in the MII stage. The development rates of MI and MII oocytes in each group were calculated (Figure 1).

### 2.4. Spindle Morphology and Classification

For evaluation of spindle morphology and classification, MI (or MII) oocytes were fixed, permeabilized, incubated with FITC-anti-α-tubulin antibody, stained with DAPI, and observed according to our previous report [26]. The spindle configuration was graded as described [47]. In brief: Grade 0 = no spindle detected; Grade 1 = severely diminished spindle, less than 50% of the normal spindle in size; Grade 2 = mildly diminished spindle, larger than 50% of the normal spindle; Grade 3 = equivalent to normal spindle size and shape (fusiform spindle); Grade 4 = equivalent to normal spindle size and shape (barrel-shaped spindle).

### 2.5. Measurement of Intracellular ROS and Glutathione (GSH) Levels

To measure intracellular ROS levels, MI (or MII) oocytes were incubated in M2 solution containing 20 µM 2, 7-dichlorodihydrofluorescein diacetate (H2DCFDA, Invitrogen, Carlsbad, CA, USA) for 30 min (37 °C, 100% humidity, and 5% CO_2_ concentration), and then washed three times in M2 solution containing 3 g/L BSA for 5 min each. Finally, oocytes were placed under a fluorescent microscope and measured under 460 nm excitation with a filter, and fluorescence images were recorded as TIFF files. After deducting background value, fluorescence intensities were quantified using Image J software (Version 1.48; National Institutes of Health, Bethesda, MD, USA) [26].

Intracellular GSH levels were measured by M2 solution containing 10 µM 4-chloromethyl-6, 8-difluoro-7-hydroxycoumarin (Cell-Tracker Blue, Invitrogen, Carlsbad, CA, USA) with a filter at 370 nm excitation, and other procedures as described for assessment of ROS, were carried out.

### 2.6. Quantitative Polymerase Chain Reaction (Q-PCR)

In each group, total complementary DNA (cDNA) was obtained from oocytes (*n* = 20–25) at MI and MII stages using TransScript-Uni Cell to cDNA Synthesis SuperMix for Q-PCR (TransGen Biotech, Beijing, China). Then, cDNA was quantified by Q-PCR using TransStart Tip Green qPCR SuperMix (TransGen Biotech, Beijing, China) on a CFX Connect Real-Time Detection System (Bio-Rad, Hercules, CA, USA) under standard conditions. Three replicates were performed for this assay, and the relative mRNA expression levels were obtained using the 2^−Δ∆Ct^ method, with *Gapdh* was used as a reference gene for normalization [48]. Primer information is detailed in Table 1.

### 2.7. Immunofluorescent Staining

The MI (or MII) oocytes were fixed in 4% (*w*/*v*) paraformaldehyde for 30 min, permeabilized in PBS with 1% Triton X-100 (*v*/*v*) for 20 min, and blocked with 1% BSA for 1 h sequentially. Then they were exposed to primary antibody at 4 °C overnight, washed three times for 5 min each in wash buffer (PBS containing 0.01% Triton X-100 and 0.1% Tween 20), and then stained with fluorescently labeled secondary antibodies and incubated at 37 °C for 1 h. After incubation, oocytes were washed three times in washing buffer for 15 min. Finally, the oocytes were placed on a clean glass slide, stained with DAPI and observed under a fluorescent microscope [49]. Slides for quantitative analysis were photographed with a fluorescent microscope under the same fluorescence parameters and magnification, and images recorded as TIFF files. Fluorescence intensities were quantified using Image J software after deducting the background value.

Antibodies and dilution ratios used in immunofluorescence were as follows: Nrf2 antibody, 1:200 (Proteintech, USA, 16396-1-AP); GCLM antibody, 1:300 (Proteintech, 14241-1-AP); HO-1 antibody, 1:300 (Proteintech, 66743-1-Ig); MEL-1A antibody, 1:200 (SC-390328, SantaCruz); and MEL-1B antibody, 1:200 (SC-398788, SantaCruz).

### 2.8. Statistical Analysis

Statistical analyses were performed by SPSS statistical software (v. 22.0; IBM, Chicago, IL, USA) to make a one-way ANOVA followed by a post hoc Fisher’s least significant difference (LSD) test. Data were expressed as the mean ± standard error and all experiments were repeated three times. For all analyses, *p* > 0.05 means no significant difference, whereas *p* < 0.05 means statistically significant.

## 3. Results

### 3.1. Melatonin Promotes In Vitro Development of Vitrified-Warmed Mouse GV Oocytes through Nrf2 Protein

The development rates of GV oocytes to MI and MII were lower (*p* < 0.05) than in the corresponding control group after vitrification (Table 2). However, when melatonin was added, the development rates of GV oocytes to MI and MII were higher (*p* < 0.05) than the corresponding vitrification group, and not different from the control groups. In contrast, when the vitrification group was co-treated with melatonin and Brusatol, the development rates of GV oocytes to MI and MII were lower (*p* < 0.05) than in the vitrification + melatonin and control groups, and there was no difference (*p* > 0.05) from the vitrification group. Therefore, melatonin promoted in vitro development of vitrified-warmed mouse GV oocytes; however, when Nrf2 protein was specifically inhibited, melatonin failed to promote maturation.

### 3.2. Melatonin Improves the Spindle Morphology Grades of MI and MII Oocytes from the Vitrified-Warmed Mouse GV Oocytes through Nrf2 Protein

**Figure 2:**
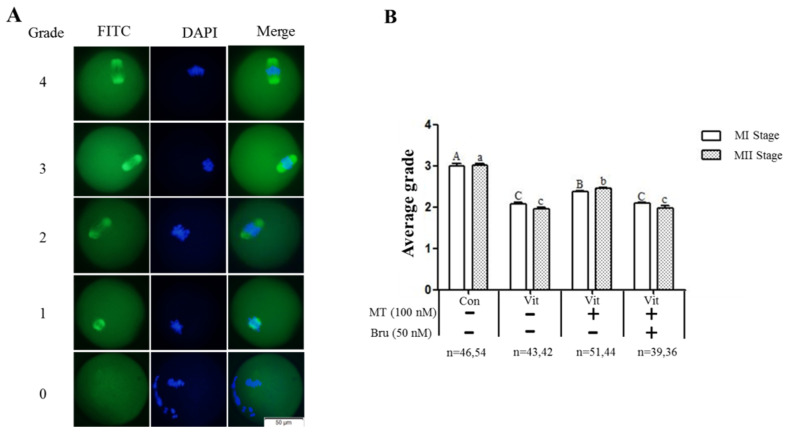
Effects of melatonin (MT) and Brusatol (Bru) on spindle morphology of MI and MII oocytes derived from vitrified-warmed GV oocytes. (**A**): Grades of spindle morphology. (**B**): Average grade of spindle morphology in four groups. Data are presented as mean ± SEM of three independent experiments. Values with different superscripts A (a), B (b), and C (c) differed (*p* < 0.05) between groups at the same stages. Scale bar = 50 µm. The four experimental groups: control (Con), vitrification (Vit), vitrification+ melatonin (Vit + MT), and vitrification + melatonin + Brusatol (Vit + MT + Bru). Notes: MI: metaphase I; MII: metaphase II.

Immunofluorescence staining of intracellular α-tubulin protein was used to observe the spindle morphology of GV oocytes as they developed to the MI and MII stages (Figure 2A). The spindle morphology grades of MI and MII oocytes in the control group were 3.00 ± 0.04 and 3.02 ± 0.04, respectively; however, the average grades of MI and MII oocytes in the vitrification group were 2.08 ± 0.03 and 1.96 ± 0.04, respectively, lower (*p* < 0.05) than the corresponding stages in the control group. With melatonin supplementation, the average grades of MI and MII oocytes were 2.37 ± 0.02 and 2.45 ± 0.03, respectively, better (*p* < 0.05) than the corresponding vitrification group, but still lower (*p* < 0.05) than the control group. However, when the vitrification group was co-treated with melatonin and Brusatol, average grades of MI and MII oocytes were 2.09 ± 0.02 and 1.97 ± 0.06, lower (*p* < 0.05) than the corresponding vitrification + melatonin and control groups, and not different (*p* < 0.05) from the vitrification group. Therefore, melatonin promoted the spindle morphology recovery of vitrified-warmed mouse GV oocytes during in vitro maturation, but when the Nrf2 protein was specifically inhibited, melatonin failed to improve spindle morphology.

### 3.3. Melatonin Regulates Oxidative Stress of MI and MII Oocytes from the Vitrified-Warmed Mouse GV Oocytes through either Nrf2 Protein or Melatonin Receptors

**Figure 3:**
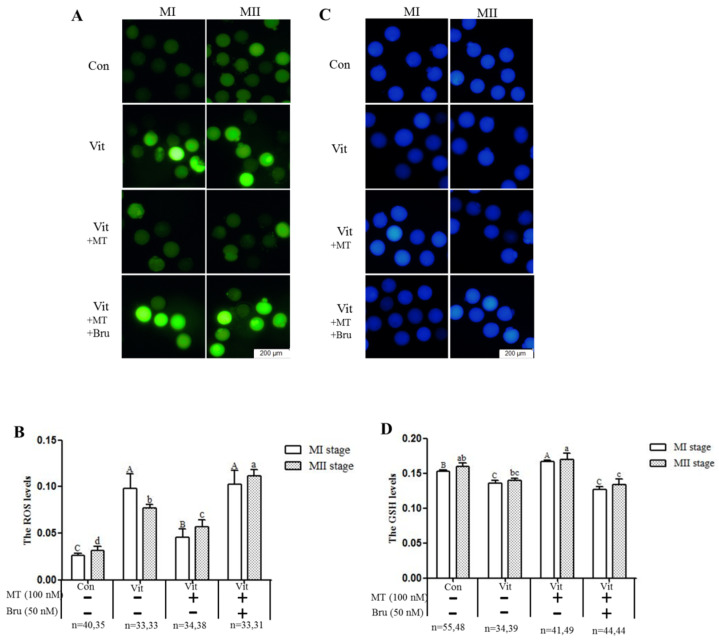
Effects of melatonin (MT) and Brusatol (Bru) on ROS and GSH levels during in vitro maturation of vitrified-warmed mouse GV oocytes. The four experimental groups had differences in (**A**,**B**) ROS levels (H2DCFDA fluorescence) and (**C**,**D**) GSH levels (Cell Tracker Blue staining). Data are expressed as mean ± SEM from three independent experiments. Different superscripts A (a), B (b), C (c), and D (d) indicate difference (*p* < 0.05) between groups at the same stages. Scale bar = 200 µm. The four experimental groups: control (Con), vitrification (Vit), vitrification+ melatonin (Vit + MT), and vitrification + melatonin + Brusatol (Vit + MT + Bru). Notes: MI: metaphase I; MII: metaphase II.

When GV oocytes developed to the MI and MII stages, ROS and GSH levels were measured to evaluate intracellular oxidative stress. The ROS levels of the MI and MII oocytes in the vitrification group were higher (*p* < 0.05) than that of the corresponding control group (Figure 3A,B). However, when melatonin was added, intracellular ROS levels (MI and MII) were lower (*p* < 0.05) than the vitrification group, but still higher (*p* < 0.05) than the control group. Furthermore, when the vitrification group was co-treated with melatonin and Brusatol, intracellular ROS levels (MI and MII) were higher (*p* < 0.05) than the corresponding vitrification + melatonin and control groups. The GSH levels of MI oocytes in the vitrification group were lower (*p* < 0.05) than in the control group (Figure 3C,D). With addition of melatonin, GSH levels of MI and MII oocytes were higher (*p* < 0.05) than the corresponding vitrification group, even at MI stage, and higher than the control group. Finally, when the vitrification group was co-treated with melatonin and Brusatol, GSH levels of the MI and MII oocytes were lower (*p* > 0.05) than the corresponding vitrification + melatonin and control groups, but not different (*p* > 0.05) from the vitrification group. Therefore, melatonin, acting through Nrf2 protein, regulated the oxidative stress level of vitrified-warmed mouse GV oocytes during in vitro development through the Nrf2 pathway.

**Figure 4:**
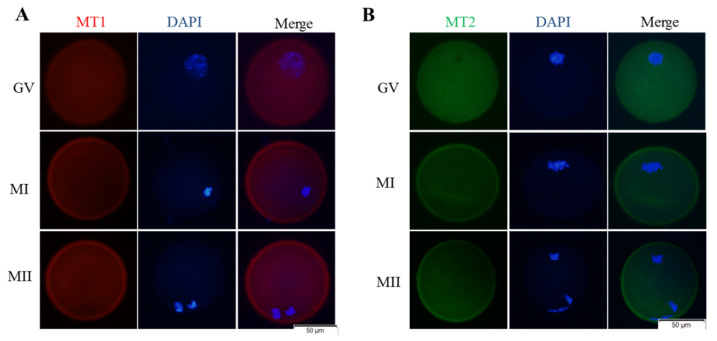
Cellular localization of MT1 and MT2 during maturation of GV oocytes. (**A**) Images of MT1 receptor (red); (**B**) Images of MT2 receptor (green). Notes: GV: germinal vesicle stage, MI: metaphase I, MII: metaphase II.

**Figure 5:**
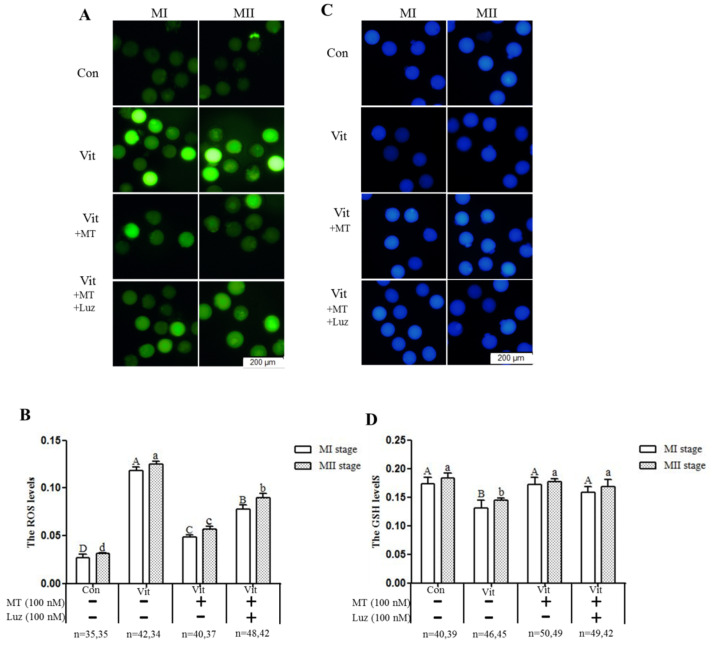
Effects of melatonin (MT) and luzindole (Luz) on ROS and GSH levels during in vitro maturation of vitrified-warmed mouse GV oocytes. (**A**): Representative fluorescence images depicting ROS intensities. (**B**): Quantification of levels of ROS in oocytes. (**C**): Representative fluorescence images depicting GSH levels. (**D**): Quantification of levels of GSH in oocytes. Data are presented as mean ± SEM of four independent experiments. Different superscripts A (a), B (b), C (c) and D (d) indicate differences (*p* < 0.05) between groups at the same stages. Scale bar = 200 µm. The four experimental groups: control (Con), vitrification (Vit), vitrification+ melatonin (Vit + MT), and vitrification + melatonin + luzindole (Vit + MT + Luz). Notes: MI: metaphase I; MII: metaphase II.

During in vitro development of vitrified-warmed mouse GV oocytes, melatonin can reduce intracellular ROS levels and increase GSH levels. Here, we further explored whether melatonin has a role through its receptors. Melatonin receptor 1 (Red, Figure 4A) and melatonin receptor 2 (Green, Figure 4B) were expressed on the membranes of GV, MI, and MII oocytes. Melatonin decreased (*p* > 0.05) ROS levels of the MI and MII oocytes after vitrification; however, when the vitrification group was co-treated with melatonin and luzindole, intracellular ROS levels (MI and MII) were higher (*p* < 0.05) than the vitrification + melatonin group, but still lower (*p* < 0.05) than the vitrification group (Figure 5A,B). The addition of melatonin increased (*p* < 0.05) intracellular GSH levels (MI and MII) after vitrification (Figure 5C,D). However, when the vitrification group was co-treated with melatonin and luzindole, there was no difference (*p* > 0.05) in GSH levels in the MI and MII oocytes compared to the corresponding vitrification + melatonin group. Therefore, during in vitro development of vitrified-warmed mouse GV oocytes, when melatonin receptors were inhibited, the ability of melatonin to scavenge ROS was reduced, but the effect on GSH levels was not significant, indicating that melatonin exerted a certain antioxidant protective role through its receptors.

### 3.4. Melatonin Regulates Nrf2 Pathway-Related Genes and Proteins of MI and MII Oocytes from the Vitrified-Warmed Mouse GV Oocytes through Melatonin Receptors

**Figure 6:**
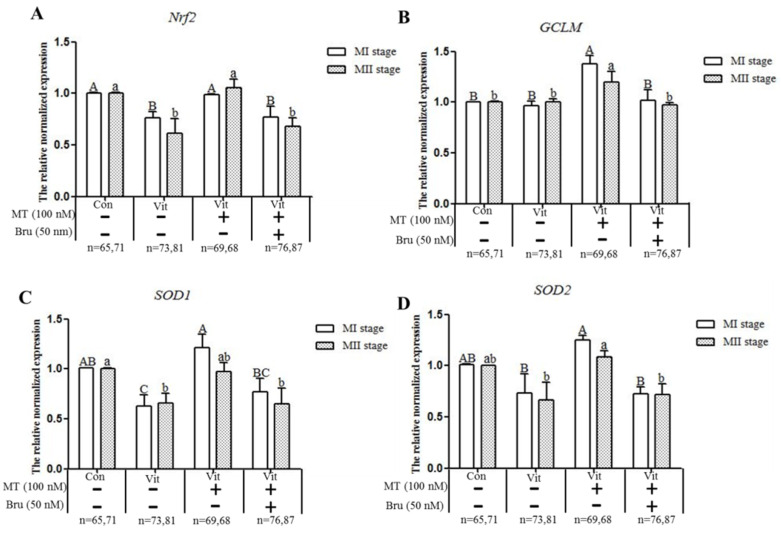
Effects of melatonin (MT) and Brusatol (Bru) on expression of Nrf2 pathway-related antioxidant genes during in vitro maturation of vitrified-warmed GV mouse oocytes. Data are presented as mean ± SEM of three independent experiments. Values with different superscripts A (a), B (b), and C (c) are different (*p* < 0.05) between groups at the same stages. Scale bar = 50 µm. The four experimental groups: control (Con), vitrification (Vit), vitrification+ melatonin (Vit + MT), and vitrification + melatonin + Brusatol (Vit + MT + Bru). Notes: MI: metaphase I; MII: metaphase II.

A qPCR analysis of the mRNA of *Nrf2*, *GCLM*, *SOD1* and *SOD2* genes during in vitro development of GV oocytes (Figure 6) produced several outcomes. When GV oocytes of the vitrification group developed to MI and MII, the expressions of *Nrf2* and *SOD1* genes were lower (*p* < 0.05) than the corresponding control group, and melatonin up-regulated (*p* < 0.05) expression of *Nrf2* and *SOD1* genes in MI oocytes and *Nrf2* gene in MII oocytes. Although vitrification did not affect (*p* > 0.05) the expression of *GCLM* and *SOD2* genes, the expressions of these two genes were increased (*p* < 0.05) after melatonin was added. When the vitrification group was co-treated with melatonin and Brusatol, except for the *SOD1* gene in MII oocytes, the expression levels of genes (*Nrf2*, *GCLM* and *SOD2*) were lower (*p* < 0.05) than in the corresponding vitrification + melatonin group. Therefore, vitrification disrupted the expression of Nrf2 signaling pathway-related antioxidant genes (*Nrf2*, *SOD1*) and in vitro development of vitrified-warmed mouse GV oocytes, although exogenous melatonin restored the expression of these genes; Finally, when the Nrf2 protein was specifically inhibited, the ability of melatonin to restore *Nrf2* and *SOD1* genes decreased.

**Figure 7:**
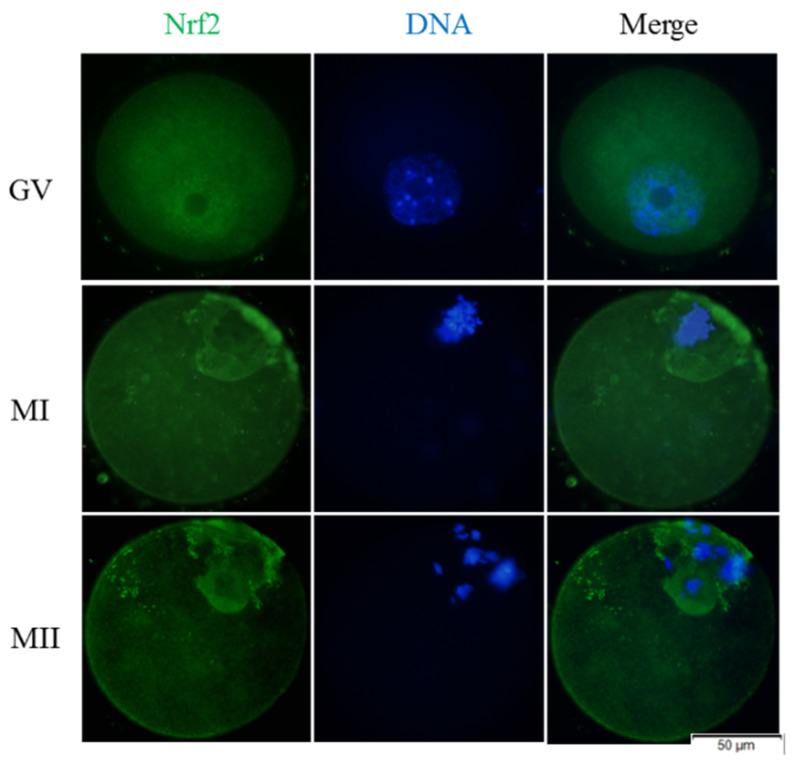
Cellular distribution of Nrf2 during oocyte meiosis. (A) Immunofluorescence staining for the subcellular localization of Nrf2 (green, Nrf2; blue, chromatin). In total 30 oocytes were examined for each group. Scale bar = 50 μm. Notes: GV: germinal vesicle, MI: metaphase I, MII: metaphase II.

**Figure 8:**
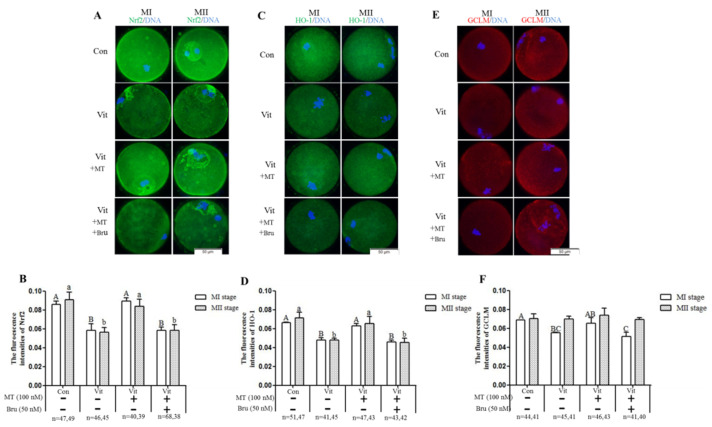
Effects of melatonin (MT) and Brusatol (Bru) on the expression of the Nrf2 pathway-related antioxidant proteins during in vitro maturation of vitrified-warmed mouse GV oocytes. Immunofluorescence of Nrf2 (**A**,**B**), HO-1 (**C**,**D**) and GCLM (**E**,**F**). Data are presented as mean ± SEM of three independent experiments. Values with different superscripts A (a), B (b), and C (c) are different (*p* < 0.05) between groups at the same stages. Scale bar = 50 µm. The four experimental groups: control (Con), vitrification (Vit), vitrification + melatonin (Vit + MT), and vitrification + melatonin + Brusatol (Vit + MT + Bru). Notes: MI: metaphase I; MII: metaphase II.

The protein expressions of Nrf2, GCLM, and HO-1 during the development of mouse GV oocytes in vitro were detected by immunofluorescence staining. We found that the Nrf2 protein was expressed in mouse GV oocytes throughout the development process in vitro (Figure 7, Green fluorescence). In GV oocytes, the Nrf2 protein was mainly located in germinal vesicles, and MI and MII oocytes were more concentrated around the chromosome. When the GV oocytes of the vitrification group developed to the MI and MII stages, the Nrf2 (Figure 8A,B) and HO-1 (Figure 8C,D) proteins were lower (*p* < 0.05) than the corresponding control group. When melatonin was added, these two proteins were higher (*p* < 0.05) than in the corresponding vitrification group, and there was no difference (*p* > 0.05) from the control group. However, when the vitrification group was co-treated with melatonin and Brusatol, these two proteins were lower (*p* < 0.05) than in the corresponding vitrification + melatonin and control groups, and there was no difference (*p* > 0.05) from the vitrification group. The GCLM protein of MI oocytes in the vitrification group (Figure 8E,F) was significantly lower than the control group. When melatonin was added, the upregulation of the GCLM protein was not significant. Furthermore, when the vitrification group was co-treated with melatonin and Brusatol, the expression of the GCLM protein in MI oocytes was lower (*p* < 0.05) than the vitrification + melatonin and control groups and there was no difference (*p* > 0.05) from the vitrification group. Furthermore, there was no difference (*p* > 0.05) among all groups in the expression of the GCLM protein in MII oocytes. Therefore, vitrification down-regulated the expression of Nrf2 and HO-1 during the development of GV oocytes in vitro, and the addition of melatonin restored the expressions of these proteins. In addition, when the Nrf2 protein was specifically inhibited, the stimulatory effect of melatonin on the HO-1 protein disappeared.

**Figure 9:**
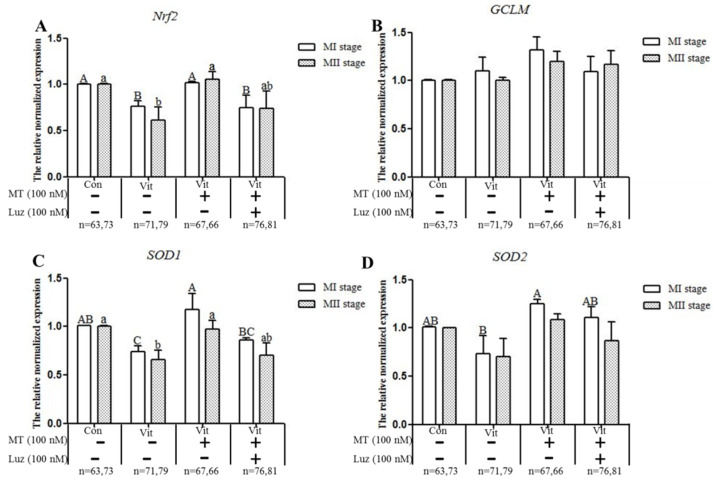
Effects of melatonin (MT) and luzindole (Luz) on the expression of Nrf2 pathway-related antioxidant genes during in vitro maturation of vitrified-warmed GV mouse oocytes. Data are presented as mean ± SEM of three independent experiments. Values with different superscripts A (a), B (b), and C (c) are different (*p* < 0.05) between groups at the same stages. Scale bar = 50 µm. The four experimental groups: control (Con), vitrification (Vit), vitrification+ melatonin (Vit + MT), and vitrification + melatonin + luzindole (Vit + MT + Bru). Notes: MI: metaphase I; MII: metaphase II.

**Figure 10:**
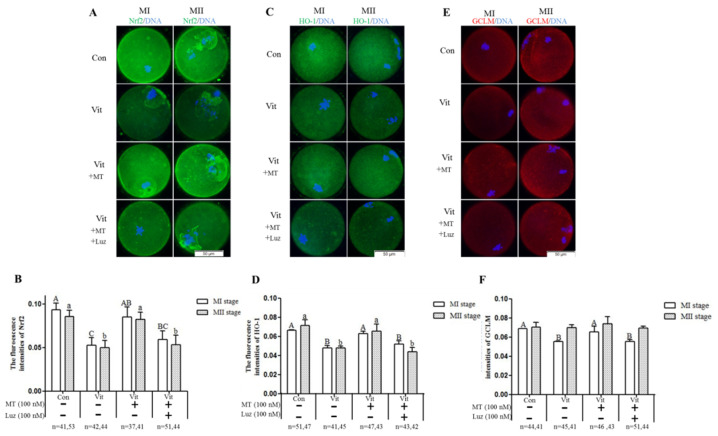
Effects of melatonin (MT) and luzindole (Luz) on the expression of Nrf2 pathway-related antioxidant proteins during in vitro maturation of vitrified-warmed mouse GV oocytes. Immunofluorescence of Nrf2 (**A**,**B**), HO-1 (**C**,**D**) and GCLM (**E**,**F**). Data are presented as mean ± SEM of three independent experiments. Values with different superscripts A (a), B (b), and C (c) are different (*p* < 0.05) between groups at the same stages. Scale bar = 50 µm. The four experimental groups: control (Con), vitrification (Vit), vitrification + melatonin (Vit + MT), and vitrification + melatonin + luzindole (Vit + MT + Lru). Notes: MI: metaphase I; MII: metaphase II.

Next, we further explored whether melatonin regulated genes (*Nrf2* and *SOD1*) and proteins (Nrf2 and HO-1) through receptors. The GV oocytes that were vitrified and co-treated with melatonin and luzindole (Figure 9) developed to the MI stage, but the gene expression levels of *Nrf2* and *SOD1* were lower (*p* < 0.05) than the vitrification + melatonin group. After the development to the MII stage, the gene expression levels of *Nrf2* and *SOD1* were not decreased (*p* > 0.05), but there was no difference (*p* > 0.05) from the vitrification group, indicating that the receptors’ inhibition also down-regulated the expression of these genes. As for the *GCLM* and *SOD2* genes, there was no difference between the co-treatment group and the only group containing melatonin at MI and MII stages. When GV oocytes were vitrified and co-treated with melatonin and luzindole (Figure 10A,B), the levels of Nrf2 proteins in the MII oocytes were lower (*p* < 0.05) than in the vitrification + melatonin and control groups. There were no (*p* > 0.05) regulation effects in the MI oocytes and no difference (*p* > 0.05) from the corresponding vitrification group in MI and MII oocytes. However, when the vitrification group was co-treated with melatonin and luzindole, levels of HO-1 protein (Figure 10C,D) in the MI and MII oocytes were lower (*p* > 0.05) than in the corresponding vitrification + melatonin and control groups, with no difference (*p* > 0.05) from the corresponding vitrification group. For the GCLM protein, the co-treatment group was lower than the melatonin group at the MI stage, but there was no difference between the two groups at the MII stage.

In summary, when melatonin receptors were inhibited, the regulatory effects of melatonin on the Nrf2 pathway-related genes (*Nrf2*, *SOD1*) and proteins (Nrf2, HO-1) during development of vitrified-warmed mouse GV oocytes were weakened, indicating that melatonin regulated the Nrf2 signaling pathway through its receptors.

## 4. Discussion

The cryopreservation of mouse [27], bovine [50], pig [51], and human oocytes [52] increases oxidative stress, although exogenous melatonin reduce oxidative stress in vitrified-warmed oocytes [30,53]. The Nrf2 signaling pathway is an important intracellular antioxidant signaling pathway, critical for maintaining redox balance [31,54]. Melatonin is a regulatory molecule of the Nrf2 protein [18]. In this study, after the Nrf2 protein was specifically inhibited by Brusatol, the potential of melatonin to reduce ROS levels decreased during in vivo development of vitrified-warmed mouse GV oocytes, and the in vitro maturation rate of oocytes was lower than that of melatonin per se. Therefore, melatonin acted on Nrf2 to regulate intracellular ROS and promote in vitro development of vitrified-warmed oocytes. Moreover, we found that the development rates and GSH levels of the Nrf2 protein-inhibited group were not significantly different from the vitrification group and the ROS levels were even higher, indicating that the function of melatonin was completely inhibited and melatonin may have played a role mainly through the regulation of the Nrf2 protein in oocytes. Moreover, melatonin can exert antioxidant protection through its receptors by antagonizing oxidative stress-induced mitochondrial dysfunction in retinal pigmented epithelium cells [55], reducing sodium fluoride-induced human hepatotoxicity [56], and improving the developmental competence of oocytes from juvenile goats [46]. In this study, when luzindole, a melatonin receptor inhibitor, was added to the vitrification group, the ability of melatonin to scavenge ROS decreased during in vivo development of vitrified-warmed mouse GV oocytes. However, it still had a significant antioxidant capacity compared to the vitrification group. Therefore, melatonin exerted a degree of antioxidative protection through the receptors in vitrified-warmed oocytes. Melatonin, as a small liposoluble molecule, easily penetrates through the cell membrane and enters into the cells, so melatonin may also enter into the oocyte directly through the osmotic pathway in order to function. High oxidative stress can cause errors in spindle formation [57,58], and melatonin can protect spindle configuration by reducing oxidative stress [26]. In this study, similar results also appeared: the vitrified-warmed mouse GV oocytes produced excessive ROS and the spindle morphology grades of MI and MII oocytes decreased during in vitro development. However, after adding melatonin, ROS decreased and spindle morphology grades recovered. In addition, when the Nrf2 protein was inhibited, the antioxidant effect of melatonin disappeared, and the spindle could not be effectively protected. Therefore, we inferred that melatonin may have acted on the Nrf2 protein to mitigate spindle damage caused by vitrification.

Cryopreservation can damage or destroy mRNA in oocytes, such as *HSP90*, *P34^cdc2^* and *Cyclin B* in sheep [59]; *CD9* in mice [60]; and *SOD1*, *Mfn2*, *Bax* and *BCL* in pigs [61]. In this study, vitrification down-regulated the expression of genes related to the Nrf2 signaling pathway (*Nrf2*, *SOD1*), which indicated that vitrification could damage the mRNA structure of related genes in the Nrf2 pathway or accelerate their degradation. Vitrification had no significant effect on *GCLM* and *SOD2* genes, indicating that these genes were more stable and perhaps either produced a strong response to cold stimulation or had differences related to the timeliness of their functions [62]. Melatonin can enhance the expression of genes related to the intracellular Nrf2 pathway in response to oxidative stress [25,35,63]. Similarly, in this study, melatonin restored levels of *Nrf2* and *SOD1* genes during in vitro development of vitrified-warmed mouse GV oocytes. Therefore, we inferred that melatonin may further activate the expression of Nrf2 signaling pathway-related genes or inhibit mRNA degradation, reduce oxidative stress, and promote in vitro maturation of vitrified-warmed mouse GV oocytes. Furthermore, melatonin can regulate the mRNA levels of Nrf2 signaling pathway-related genes through receptors in response to damage [38,64]. In this study, after melatonin receptors were inhibited, the mRNA levels of *Nrf2* and *SOD1* genes in the MI oocytes were significantly decreased, and *Nrf2* and *SOD1* mRNA levels in MII oocytes were also down-regulated. Therefore, melatonin can also regulate genes related to the Nrf2 signaling pathway through receptors during in vitro maturation of vitrified-warmed mouse GV oocytes.

Cryopreservation can destroy some proteins in oocytes, e.g., porcine Nrf2, human aquaporin 1 (APQ1) [31], goat CDK1 [65], and mouse Mad2 [66], etc. After the vitrification of porcine COC oocytes, proteomics analysis revealed that 59 proteins were down-regulated [67]. Similarly, in this study, the vitrification of mouse GV oocytes resulted in a significant down-regulation of Nrf2 and the downstream antioxidant protein HO-1 was disturbed in MI and MII during in vitro maturation. The disorder of protein expression caused by cryopreservation may be directly caused by damage to the protein structure, or lead to the degradation of the corresponding mRNA, which ultimately affects basal protein levels.

During in vitro maturation of vitrified-warmed mouse GV oocytes, 10^−7^ M melatonin restored the expression levels of Nrf2 and HO-1, indicating that melatonin can reduce the intracellular oxidative stress by protecting proteins related to the Nrf2 signaling pathway. Similarly, melatonin-enhanced Nrf2 and downstream proteins in response to injury stress on porcine COC oocytes [35], rat hippocampus [68], human alveolar epithelial cells [23], and rat bladders [69], etc. Melatonin can regulate Nrf2 signaling pathway-related proteins to respond to stress through its receptors [38,39]. In this study, when melatonin receptors were inhibited, melatonin could not effectively restore the expression of Nrf2 and downstream HO-1 during in vitro development of vitrified-warmed GV oocytes, which indicated that melatonin receptors were nodes of melatonin’s antioxidant mechanism. This was apparently the first report that melatonin regulated the Nrf2 signaling pathway through the receptors of vitrified-warmed mouse oocytes.

Based on the above, we speculate that during the in vitro maturation of vitrified-warmed mouse GV oocytes, melatonin may exert an antioxidant role through the MT1/MT2-Nrf2 pathway. Similarly, melatonin can also enter oocytes through osmosis, because when the receptors are completely inhibited, the regulation of melatonin on Nrf2 pathway-related genes and proteins is partially reduced. Either way, the Nrf2 protein is an important node of melatonin action.

## 5. Conclusions

**Figure 11:**
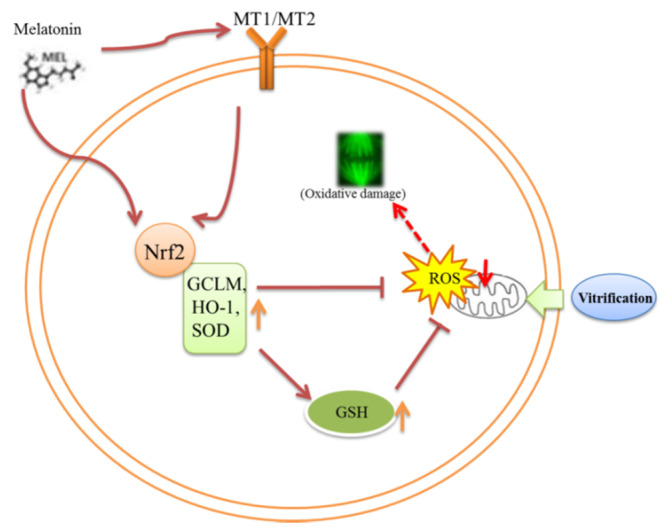
A schematic illustration of the cross talk between the Nrf2 signaling pathway activated by melatonin in vitrified-warmed mouse GV oocytes during in vitro maturation. MT1/MT2: melatonin receptors. Nrf2, GCLM, HO-1, SOD: antioxidant protein. GSH: glutathione. ROS: reactive oxygen species.

Vitrification of mouse GV oocytes could alter the expression of Nrf2 signaling pathway-related genes (*Nrf2*, *SOD1*) and proteins (Nrf2, HO-1), disrupt the redox system, damage spindle structure, and decrease oocyte development potential during in vitro maturation. The addition of 10^−7^ M melatonin may act on the Nrf2 signaling pathway through its receptors, promote the expression levels of genes and proteins to return to normal, reduce the oxidative stress and spindle damage of oocytes induced by vitrification, and increase the in vitro maturation rate of oocytes (Figure 11).

## Figures and Tables

**Table 1:** Primers for genes.

Gene	Accession Number	Primer Sequence (5′–3′)	Tm (°C)
*Nrf2*	NM_010902.4	F:GTCTTCACTGCCCCTCATC R:TCGGGAATGGAAAATAGCTCC	55
*Gclm*	NM_008129.4	F:CTTGGAGCATTTACAGCCTTAC R:GTGAGTCAGTAGCTGTATGTCA	55
*Sod1*	NM_011434.2	F:CACTCTAAGAAACATGGTGG R:GATCACACGATCTTCAATGG	55
*Sod2*	NM_013671.3	F:AAGGGAGATGTTACAACTCAGG R:GCTCAGGTTTGTCCAGAAAATG	55
*Gapdh*	NM_008084.3	F:CATGGCCTTCCGTGTTCCTA R:GCCTGCTTACCACCTTCTT	55

**Table 2:** In vitro development of vitrified mouse GV oocytes after melatonin and Brusatol treatment.

Group	Melatonin (mol/L)	No. GV Oocytes Vitrified	No. GV Oocytes Recovered	No. GV Oocytes with Normal Morphology for (MI, MII)	No. GV Oocytes That Matured to	
					MI Stage at 9 Hpi (%)	MII Stage at 13 Hpi (%)
C	0			60, 73	46(76.87 ± 1.82) ^a^	57(77.03 ± 1.97) ^a^
V	0	170	164	72, 73	43(59.42 ± 2.44) ^b^	41(56.92 ± 3.05) ^b^
V + M	10^−7^	151	145	65, 63	48(73.95 ± 0.53) ^a^	49(72.62 ± 2.03) ^a^
V + M + B	10^−7^	155	147	64, 63	39(59.85 ± 4.07) ^b^	35(56.01 ± 2.01) ^b^

Fresh GV oocytes were randomly divided into four groups: control (Con), vitrification (Vit), vitrification + melatonin (Vit + MT), and vitrification + melatonin + Brusatol (Vit + MT + Bru). Oocytes were evaluated to be morphologically normal by visual inspection of membrane integrity, zona pellucida (ZP), and any altered appearance of cytoplasm (e.g., becoming white, colorless, or dispersed). The percentage of MI oocytes and MII oocytes were calculated from the number of oocytes with normal morphology. Values with different superscripts (^a^ and ^b^) in the same are significantly different *(p* < 0.05). Notes: GV: germinal vesicle; MI: metaphase I; MII: metaphase II; hpi: hours post in vitro culture.
